# Microbial community differentiation in vent chimneys of the Lost City Hydrothermal Field reflects habitat heterogeneity

**DOI:** 10.3389/frmbi.2024.1401831

**Published:** 2025-01-20

**Authors:** Osama M. Alian, William J. Brazelton, Karmina A. Aquino, Katrina I. Twing, H. Lizethe Pendleton, Gretchen Früh-Green, Susan Q. Lang, Matthew O. Schrenk

**Affiliations:** ^1^ Department of Microbiology, Genetics, and Immunology, Michigan State University, East Lansing, MI, United States; ^2^ School of Biological Sciences, University of Utah, Salt Lake City, UT, United States; ^3^ Department of Science and Technology - Philippine Nuclear Research Institute, Quezon City, Philippines; ^4^ Department of Microbiology, Weber State University, Ogden, UT, United States; ^5^ Department of Earth Sciences, Eidgenössiche Technische Hochschule Zürich, Zürich, Switzerland; ^6^ Department of Geology and Geophysics, Woods Hole Oceanographic Institution, Woods Hole, MA, United States; ^7^ Department of Earth and Environmental Science, Michigan State University, East Lansing, MI, United States

**Keywords:** hydrothermal vent, geomicrobiology, correlations, microbe-rock interactions, extremophile, Lost City Hydrothermal Field

## Abstract

Oceanic hydrothermal vent systems represent some of the oldest habitats on Earth and serve as analogs for extraterrestrial environments. The Lost City Hydrothermal Field (LCHF) near the Mid-Atlantic Ridge is one such environment, and its large chimneys are unique in hosting actively venting hydrothermal fluids that are primarily controlled by serpentinization reactions in the subseafloor. Microbial communities within LCHF have been studied for insights into their functional adaptations to the warm, alkaline, and dissolved inorganic carbon-limited environment. Metagenomic and mineralogical data collected during a recent expedition to Lost City were analyzed to delineate associations between microbial populations and physical, chemical and biological characteristics of the chimneys. Bacterial 16S rRNA gene sequences show a high degree of putative microdiversity within the relatively dominant genera Desulfotomaculum, Sulfurovum, Thiomicrorhabdus, and Serpentinicella, which represent a large core of the overall LCHF vent bacterial community. This microdiversity relates to the compositional fraction of aragonite, brucite, and calcite minerals within chimney samples rather than just the composition of nearby vent fluids. Although many species are found in both chimneys and venting fluids, the overall microbial community structures in chimney biofilms remain distinct from the hydrothermal fluids that flow through them. Shotgun metagenomic analyses reveal differences among genes predicted to be involved in carbon, methane, nitrogen and sulfur cycling with respect to their correlations to the abundances of specific minerals. These data hint at microenvironmental complexity lost within standard bulk analyses. The findings of this study underscore the need to more closely examine microbe-mineral interactions in natural environments, critically informing not just population-level distributions, but also the functional underpinnings of these extremophile microbial communities.

## Introduction

Deep-sea hydrothermal vents are some of the most dynamic environments in Earth’s biosphere. These unique ecosystems form when hydrothermal fluids, produced from processes below the seafloor, mix and react with cold, oxic seawater (Corliss et al., 1979; Früh-Green et al., 2022). Specific niches at deep-sea vents contain some of the harshest conditions known to support life, with extreme temperatures (>120°C), pressures (> 50 MPa) and pH (from 3-10) (Ding et al., 2005; Ludwig et al., 2006). Vent chimneys represent distinct habitats within marine hydrothermal ecosystems, serving as conduits for fluid flow as well as platforms for sustainable chemical disequilibria between the ocean and subseafloor (Sander and Koschinsky, 2011; Resing et al., 2015). The inhabitants of vent ecosystems redefine our understanding of biological resilience and the limits of habitability in extreme settings. Microbial communities found here often have unique taxonomic diversity, containing organisms with physiologies adapted to the distinctive physical-chemical conditions of the vents (Nakagawa and Takai, 2008; Anderson et al., 2015; Jebbar et al., 2015; Meier et al., 2017). Within the porous walls of hydrothermal chimneys, steep thermal and chemical gradients similar to those found in the subseafloor develop over distances as short as a few centimeters (Kristall et al., 2006). Although the microbiological features of vent chimneys have been previously studied at larger scales, limited research has been conducted on the characteristics of small-scale micro-environments within the chimney walls and how they influence microbial community organization and functions (Wang et al., 2009). Prior studies delineate microbial distributions at coarse scales, highlighting differences between the inner and outer portions of chimneys or between different vent habitats (Harmsen et al., 1997; Takai et al., 2001; Schrenk et al., 2003; Scott et al., 2017). However, the nuanced distribution and abundance of these communities across varying local conditions, exemplified by different mineralogy, remain to be fully understood.

Sulfide-hosted hydrothermal systems, driven by active magmatism, are among the best studied extreme environments since their discovery in the 1970s. These systems are teeming with uniquely adapted microbial and macrofaunal communities, some of which endure the highest temperatures known to life (Prieur, 1997; Gadanho and Sampaio, 2005; Wirth, 2017). A mix of seawater-derived nutrients and oxidants, combined with metals and volatiles from magmatic processes, sustain high microbial metabolic activity within these vibrant ecosystems (Kato et al., 2012; Zhong et al., 2022). In contrast, low to moderate temperature hydrothermal systems influenced by serpentinization, a process whereby water reacts with ultramafic rocks of the upper mantle, can be metal-deficient with scant dissolved inorganic carbon and high pH from the water-rock reactions (Ludwig et al., 2006). The Lost City Hydrothermal Field (LCHF; 30°N, Mid-Atlantic Ridge) is an example of this type of system, and vents alkaline, warm fluids. The chimney structures at LCHF consist primarily of three minerals; aragonite (CaCO_3_), brucite (Mg(OH)_2_) and calcite (CaCO_3_). These minerals form as the emanating serpentinization-influenced fluids react with seawater (Chavagnac et al., 2013; Tutolo et al., 2018; Aquino and Früh-Green, 2024a; Aquino and Früh-Green, 2024b). Studies of LCHF chimneys indicate major controls of vent fluid and seawater mixing on the resulting mineralogy. Brucite-calcite is the preferred mineral assemblage that precipitates from the vent-dominated solutions found in interior chimney structures, with limited seawater mixing (Aquino et al., 2023; Aquino and Früh-Green, 2024b). In contrast, chimney exteriors in a seawater-dominated environment are primarily aragonite. These differences are further highlighted by stable isotope signatures suggesting higher formation temperatures in the brucite-calcite regions and ^87^Sr/^86^Sr ratios in aragonite that are much closer to seawater than primary calcite. As venting decreases and the chimneys become extinct, structures develop much larger fractions of secondary calcite infilling than either aragonite or brucite (Ludwig et al., 2011). Taken together, these minerals can be used as proxies for environmental conditions within chimney structures where brucite represents the hottest and newest chimneys, aragonite appears with increasing seawater intrusion, and the largest relative calcite fractions indicate terminal stage vents.

The fragile nature of LCHF chimneys, sampling challenges associated with this remote site, and difficulty culturing representative microbial populations have limited our ability to investigate the microbiology of the chimneys. Prior work has shown that the chimneys host dense microbial populations, reaching 10^9^ cells per gram, primarily as biofilms (Brazelton et al., 2011). Although the serpentinization-influenced hydrothermal fluids at LCHF provide key energy sources such as methane, hydrogen and formate, they are largely deficient in trace metals that are critical to enzymatic functions (Brazelton et al., 2006; Ludwig et al., 2006; Brazelton et al., 2022). The presence of dense microbial biofilms within these chimneys hint at sufficient nutrient availability to sustain metabolism in either the fluids or the chimney minerals, or an adaptation to utilize alternate metabolic cofactors.

Metal limitations can greatly impact microbial physiology and the functional organization of microbial communities. Metal cofactors are required for many metabolic processes, including methane and nitrogen cycling, and most basic cell functions, such as electron transport. They are especially critical to key steps in anaerobic metabolism that under thermophilic conditions, can require nearly double (hundreds of ppm and ppb levels) the trace metal amounts for mesophilic conditions (Takashima et al., 2011). Lost City vent fluids and carbonates contain generally lower metal concentrations than seawater (Kelley et al., 2005; Ludwig et al., 2006; Früh-Green et al., 2022). When chimneys are analyzed in bulk, constituent trace elements are generally minimal or below instrument detection depending on the element of interest, with difficulties in detectability arising from mineral matrix effects (Ludwig et al., 2006). In other systems, metal concentrations can vary over time with changes in the underlying vent fluid chemistry, temperature and pH (Schijf et al., 2015; Zwicker et al., 2022). Marine microorganisms inhabit a wide range of metal-scarce environments and have evolved sophisticated adaptive strategies for metal uptake, or evolved to use alternative cofactors altogether (Vasudevan et al., 2002; Samuel et al., 2012; Boyd et al., 2015; Fadel et al., 2017; Mus et al., 2019). These communities may also engage in cooperative strategies, sharing resources and products in a complex biofilm community (Liang et al., 2017; Skennerton et al., 2017). Effective utilization of these trace metals likely depends on the existence of localized microenvironments and mixing regions at the mineral-microbe interface (McCollom and Shock, 1997; Bianchi et al., 2018).

This study examines the diversity and differentiation of microbial populations within the Lost City chimneys and how they relate to physical-chemical features and chimney evolution. We highlight how specific taxa and genes are associated with the key indicator minerals aragonite, brucite and calcite, which are used as proxies for localized conditions. Our findings shed light on the interplay between geochemistry and biochemistry within these structures, identifying small scale environmental differences that may impact microbial community structure within the Lost City chimneys and delineating some of the key metabolic pathways prevalent in those environments.

## Materials and methods

### Sample collection

Hydrothermal chimney and fluid samples were collected using the R/V Atlantis and ROV Jason II during expedition AT42-01 in September 2018. Hydrothermal fluids and seawater were sampled with the Hydrothermal Organic Geochemistry sampler deployed on ROV Jason II or with Niskin bottles mounted to a CTD rosette, as previously described (Lang and Benitez-Nelson, 2021; Lang et al., 2021; Brazelton et al., 2022). Water samples were also collected with Niskin bottles mounted to a CTD rosette and Niskin bottles mounted to two seafloor drills during IODP Expedition 357 (Früh-Green et al., 2018; Motamedi et al., 2020). Hydrothermal fluid samples collected with ROV Jason II were filtered *in situ* through 0.22 µm Sterivex filter cartridges (Millipore, Billerica, Massachusetts, USA). Niskin water samples were filtered shipboard through 0.22 µm Sterivex filter cartridges using an inline peristaltic pump. Sterivex filter cartridges were stored frozen at –80°C until analysis in the shore-based laboratory, as previously reported (Motamedi et al., 2020; Brazelton et al., 2022). Ambient lab air samples were collected from the R/V Atlantis shipboard laboratory and at the University of Utah during DNA extraction procedures, as we have reported for other studies (Motamedi et al., 2020; Brazelton et al., 2022).

Chimney samples were collected by slurp sampling from the ROV or via grab sampling and placed in onboard boxes unique to each item. Samples were processed aseptically with sterile tools shipboard shortly after collection. Subsamples of various sizes and masses were collected shipboard and either stored in sterile Falcon tubes or Whirlpaks to preserve integrity. Each individual sample is associated with a unique longform Jason dive ID to track any associated parallel analyses performed. Collections were catalogued to record location information, depth, pH and temperature where measured and any notes during sampling. Samples destined for genomics work were immediately frozen at -80°C on the ship and transferred by dry ice to the University of Utah for DNA extraction.

### X-ray diffraction mineralogy analysis

The mineralogy of the samples was determined using a Bruker AXS D8 Advance Powder X-ray Diffractomer (Bruker Corporation, Billerica, USA) at the Institute of Geochemistry and Petrology, ETH Zurich. The XRD was equipped with a LynxEye detector and a CuKα source. The analyses were carried out using a scan range of 5-90° 2θ, a step size of 0.02, and a scan time of 0.8 seconds per step. The mineral phases were identified using the ICDD PDF-2 database (International Center for Diffraction Data, USA) and the ICDD Sieve+ automatic peak search software. Weight proportions of the identified minerals were obtained using a full-profile Rietveld refinement method via Siroquant version 3.0 (Seitronics, Australia). Detailed mineralogical studies were carried out by Aquino et al. and are reported in a separate publication (Aquino and Früh-Green, 2024a; Aquino and Früh-Green, 2024b)

### DNA extraction and sequencing

Extractions of DNA from chimney biofilm samples were conducted with the FastDNA SPIN kit (MP Biomedical) according to the manufacturer’s instructions, followed by washing in Amicon Ultra (Millipore) filter units with 65°C Tris-EDTA buffer. Extractions of DNA from hydrothermal fluid samples and seawater samples were conducted as described previously (Motamedi et al., 2020; Brazelton et al., 2022) and the full extraction protocol is available on protocols.io (DOI: dx.doi.org/10.17504/protocols.io.5qpvoym5xg4o/v2). All DNA preparations were purified with magnetic beads prior to sequencing (Rohland and Reich, 2012).

Sequencing of 16S rRNA gene amplicons was conducted by the Michigan State University genomics core facility. The V4 region of the 16S rRNA gene was amplified with universal dual-indexed Illumina fusion primers (515F/806R) as described elsewhere (Wang et al., 2009). Amplicon concentrations were normalized and pooled using an Invitrogen SequalPrep DNA Normalization Plate. After library quality control (QC) and quantitation, the pool was loaded on an Illumina MiSeq v2 flow cell and sequenced using a standard 500 cycle reagent kit. Base calling was performed by Illumina Real Time Analysis (RTA) software v1.18.54. Output of RTA was demultiplexed and converted to FASTq files using Illumina Bcl2fastq v1.8.4.

Chimney biofilm metagenomic libraries were constructed with size-selected, sonicated DNA fragments of 500-700 bp with the NEBnext Ultra DNA II library kit for Illumina (E7645S). Paired-end sequencing (2 × 150 bp) of metagenomic libraries was conducted at the University of Utah High-Throughput Genomics Core Facility at the Huntsman Cancer Institute with an Illumina NovaSeq 6000 platform.

16S rRNA gene amplicon sequences were processed with cutadapt v. 1.15 and DADA2 v. 1.10.1, as in previous studies (Motamedi et al., 2020; Brazelton et al., 2022). This protocol includes quality trimming and filtering of reads, removal of chimeras, and inference of amplicon sequence variants (ASVs). Taxonomic classification of all ASVs was performed with DADA2 using the SILVA reference alignment (SSURefv132) and taxonomy outline. Sequences from the same sequencing run were analyzed together with DADA2, and the resulting ASVs from separate sequencing runs were merged with phyloseq (McMurdie and Holmes, 2013). The merged set of ASVs included sequences from chimney biofilms (present study), hydrothermal fluids (Brazelton et al., 2022), seawater (Motamedi et al., 2020; Lang et al., 2021; Brazelton et al., 2022), seafloor rock drill cores (Motamedi et al., 2020), ambient lab air [present study and (Motamedi et al., 2020; Brazelton et al., 2022)], and extraction blanks [present study and (Motamedi et al., 2020; Brazelton et al., 2022)]. Potential contaminants were removed from the merged set of ASVs with the decontam package using both the “prevalence” and “frequency” modes, as previously reported (Brazelton et al., 2022). A total of 53 ASVs representing 1,856 sequence counts were removed from chimney biofilm samples with this approach. In addition, ASVs with taxonomic assignments matching those identified as common contaminants of subsurface sequence datasets (Sheik et al., 2018) were removed from downstream analyses, resulting in the removal of an additional 422 ASVs. Finally, ASVs from chloroplasts and mitochondria were filtered from the data set based on taxonomic assignment.

### Metagenomic sequencing assembly and annotation

Metagenomic analyses were conducted as described previously for a similar study (Brazelton et al., 2022) and summarized here. Adapters and low-quality bases were removed from metagenomes with bbduk and seq-qc (Bushnell et al., 2017; Thornton et al., 2020). A pooled, co-assembly of all 16 chimney biofilm metagenomes was conducted with Megahit v1.1.1 (Li et al., 2016). Genes were predicted with Prodigal v2.6.3 in meta mode (Hyatt et al., 2010). Predicted protein sequences were queried against the KEGG release 83.2 prokaryotes database with Diamond v0.9.14 (Buchfink et al., 2021). Unassembled sequences were mapped to the pooled Megahit assembly with Bowtie2 v2.3.2 (Langmead and Salzberg, 2012). Binning of metagenome-assembled genomes (MAGs) from the pooled assembly was conducted with BinSanity using the Binsanity-lc workflow v0.2.6.2 and a minimum contig size of 3 kb (Graham et al., 2017). MAGs were assigned taxonomic classifications with GTDB-Tk v1.5.1 (reference data version r202 (Chaumeil et al., 2019);). Completeness and contamination of MAGs were estimated with CheckM v1.0.5 (Parks et al., 2015). The coverage for each predicted protein and KEGG annotation feature was calculated as transcripts (or fragments) per million (TPM) with count_features v1.3.0, part of our seq-annot package (Thornton et al., 2020; Brazelton et al., 2022). TPM is a proportional unit (multiplied by one million for convenience) that is normalized to the length of each predicted protein sequence as well as to the total library size. The coverage of each MAG was calculated as the weighted sum of the normalized, proportional coverages (in TPM) of its member contigs. The contig coverages were obtained by mapping all unassembled reads against each MAG with Bowtie2 and then calculating the average coverage per position of each contig with the genomecov command (with the option -pc) in bedtools v2.30.0 (Quinlan and Hall, 2010). Normalized coverages in units of TPM were calculated by dividing the average coverage per position by the total number of read pairs for that library. Metagenomic bins used for this study are summarized in **Supplementary Table 1**.

### Statistical analyses of amplicon and metagenomic data

16S rRNA data was imported as ASVs and analyzed using the R studio environment version 4.2.2, primarily utilizing the Phyloseq package (McMurdie and Holmes, 2013; R Core Team, 2022). NMDS analyses were done within Phyloseq using the ordinate() function on Bray-Curtis dissimilarities of ASV relative abundances. ASV counts were normalized to the median sequence depth and transformed to proportional abundance. Source-tracking to identify potential sources and sinks of sequences was carried out using the R package FEAST (Shenhav et al., 2019). Samples of seawater collected during the 2018 Lost City expedition, as well as IODP Expedition 357, were classified as putative sources, while plume, biofilm and vent fluids were categorized as sinks (Lang et al., 2021). FEAST outputs assigned a fractional contribution between labeled sources and sinks, categorizing unknown contributions as their own category. Pearson correlation tests were carried out in the R environment using the ltm package and the rcor.test() function for the ASV-mineralogy matrix and the genes-mineralogy matrix (Rizopoulos, 2007). A correction for multiple tests was carried out using the R package qvalue and qvalue() function (Storey, 2002; Storey and Tibshirani, 2003). Significant correlation scores (p < 0.05 and q < 0.05) were retained as the statistically significant output for further analysis. Accordingly, three groups of correlations were analyzed within these constraints: aragonite (ARA), brucite (BRU) and calcite (CAL), based on what mineral they were strongly correlated to.

Metagenome Assembled Genomes (MAGs) with over 90% completion and less than 10% contamination were tested for their mineralogical Pearson correlations in a pairwise matrix as before by using their calculated sequence coverage (in TPM). Bin metabolic potential was assessed by the presence and completeness of KEGG metabolic modules for carbon fixation, nitrogen cycling, methane cycling and sulfur cycling, in addition to a selected subset of metal cycling or environmental processing genes (Kanehisa and Goto, 2000).

## Results

### Chimney mineralogy and community characteristics

Chimney samples were collected from seven sites across the LCHF at depths between 729 and 875 meters below sea level (**Table 1**). Aragonite and brucite were the dominant minerals, but sample lithologies were usually a combination of all three minerals. Temperature, pH and chemistry of associated hydrothermal fluids were concurrently measured during the same expedition, indicating temperatures at the site up to 96°C, *in situ* pH up to 10, and trends in chemistry consistent with the mixture of H_2_-rich hydrothermal fluids with ambient seawater (Aquino et al., 2022; Brazelton et al., 2022).

**Table 1:** Samples included in this study, their location, label used for analysis, and mineralogical content for aragonite, brucite and calcite in weight percent.

Sample	Jason Sample ID	Location	Location Label	Type	Depth (m)	Latitude	Longitude	Aragonite (wt%)	Brucite (wt%)	Calcite (wt%)
LC02867	J.1107.16Sept.1708	Marker 3	MKR3	Active Chimney	729.67	30.1205243	-42.1243348	30	66	3
LC02870 A/C	J.1107.16Sept.1723	Marker 3	MKR3	Active Chimney	729.69	30.1205071	-42.1243435	20	78	2
LC02876	J.1107.16Sept.1714	Marker 3	MKR3	Active Chimney	729.7	30.1205091	-42.1243436	35	61	4
LC02881	J.1107.16Sept.1713	Marker 3	MKR3	Active Chimney	729.68	30.1205119	-42.1243414	50	49	1
LC02923	J.1108.17Sept.2301	Marker 6	MKR6	Active Chimney	797.97	30.1245919	-42.1193596	65	34	1
LC02928	J.1108.17Sept.2002	Calypso	CAL	Active Chimney	797.95	30.1245946	-42.1193284	23	54	22
LC02934	J.1108.17.Sept.2223	Venting Wall	VW	Venting Wall	795.19	30.1246195	-42.1193754	94	6	1
LC02938	J.1108.17Sept.1338	Calypso	CAL	Active Chimney	797.44	30.1242902	-42.1193738	26	71	2
LC02954	J.1109.Sep19.0756	Sombrero	SOM	Active Chimney	761.71	30.1240556	-42.1195502	96	4	1
LC02958	J.1109.Sep19.1033	Carbonate Veins	CV	Inactive Vein	741.1	30.1249291	-42.1188567	13	59	28
LC02964	J.1109.Sep19.1011	Carbonate Veins	CV	Inactive Vein	740.89	30.1249447	-42.1188485	29	42	29
LC02967	J.1109.Sep19.1016	Carbonate Veins	CV	Inactive Vein	740.9	30.1249298	-42.1188394	47	24	29
LC02981	J.1110.20Sep.0611	Marker 8	MKR8	Active Chimney	801.21	30.1204738	-42.1249175	30	68	1
LC02985	J.1110.20Sep.0609	Marker 8	MKR8	Active Chimney	801.21	30.1204738	-42.1249175	34	63	2
LC02990	J.1110.20Sep.0817	Marker 8 Vein	MKR8V	Inactive Vein	871.44	30.1203248	-42.1257789	14	61	25
LC02993 B/D	J.1110.20Sep.0838	Marker 8 Vein	MKR8V	Inactive Vein	874.96	30.1202751	-42.1256963	38	18	33
LC02998	J.1111.21Sep.0938	Marker 6 Wall	MKR6W	Inactive Vein	788.23	30.1177429	-42.1247982	94	1	6
LC03001	J.1111.20Sep.2240	Sombrero	SOM	Active Chimney	762.11	30.119726	-42.124407	62	32	6
LC03005	J.1111.20Sep.2214	Sombrero	SOM	Active Chimney	762.08	30.1197175	-42.1243882	68	24	9
LC03008	J.1112.22sSep.0113	Marker 6	MKR6	Active Chimney	777.05	30.1207253	-42.1241496	87	12	2
LC03012	J.1112.22Sep.0110	Marker 6	MKR6	Active Chimney	777.16	30.1207231	-42.1241433	51	49	1

Examining chimney microbial communities through their ASV composition reveals only moderate clustering of samples collected from the same chimney, such as in Marker 3 samples (**Supplementary Figure 1**). This heterogeneity is in line with previous observations of physical variability in venting dynamics and mineral composition even across short distances within one chimney structure (Schrenk et al., 2004; Kelley et al., 2005; Ludwig et al., 2006). Microbial species richness is also variable among samples from the same chimney, with MKR3 and MKR8 samples having the lowest richness estimates (**Supplementary Figure 2**). Nearly 50% of all sequence counts in the dataset are dominated by just 50 ASVs constituting 14 genera (**Supplementary Figure 3**; **Table 2**). Omitting unassigned taxa, the most abundant genera show high numbers of unique ASVs, the largest being *Thiomicrorhabdus* with 111 ASVs, indicating a large contribution of sequence diversity from relatively few taxa, consistent with earlier studies (Brazelton et al., 2011).

**Table 2:** Representative table of the most abundant genera sequenced from the vents in order of decreasing total percent relative abundance, and including the number of ASVs found in our dataset.

Genus	# Unique ASVs	% Abundance
*Thiomicrorhabdus*	111	8.9
*Desulfotomaculum*	50	8.3
*Roseobacter_clade_NAC11-7_lineage*	17	7.9
*Sulfurovum*	74	7.1
*Marine_Methylotrophic_Group_2*	37	3.5
*Sulfurospirillum*	15	2.8
*Cocleimonas*	33	2.6
*Serpentinicella*	20	2.3
*pItb-vmat-59*	24	2
*Moritella*	4	1.7
*Marine_Methylotrophic_Group_3*	19	1.7
*Sva0996_marine_group*	70	1.4
*IheB2-23*	47	1.3
*Desulfobulbus*	9	0.9
*Actibacter*	18	0.8
*Methanosalsum*	7	0.7
*Sedimenticola*	4	0.4
*Lutimonas*	6	0.4
*Woeseia*	85	0.4
*Ruegeria*	2	0.4
*Desulfurivibrio*	3	0.4
*Filomicrobium*	46	0.4
*Litoreibacter*	3	0.3
*Cenarchaeum*	2	0.3
*Photobacterium*	4	0.3

**Figure 1:**
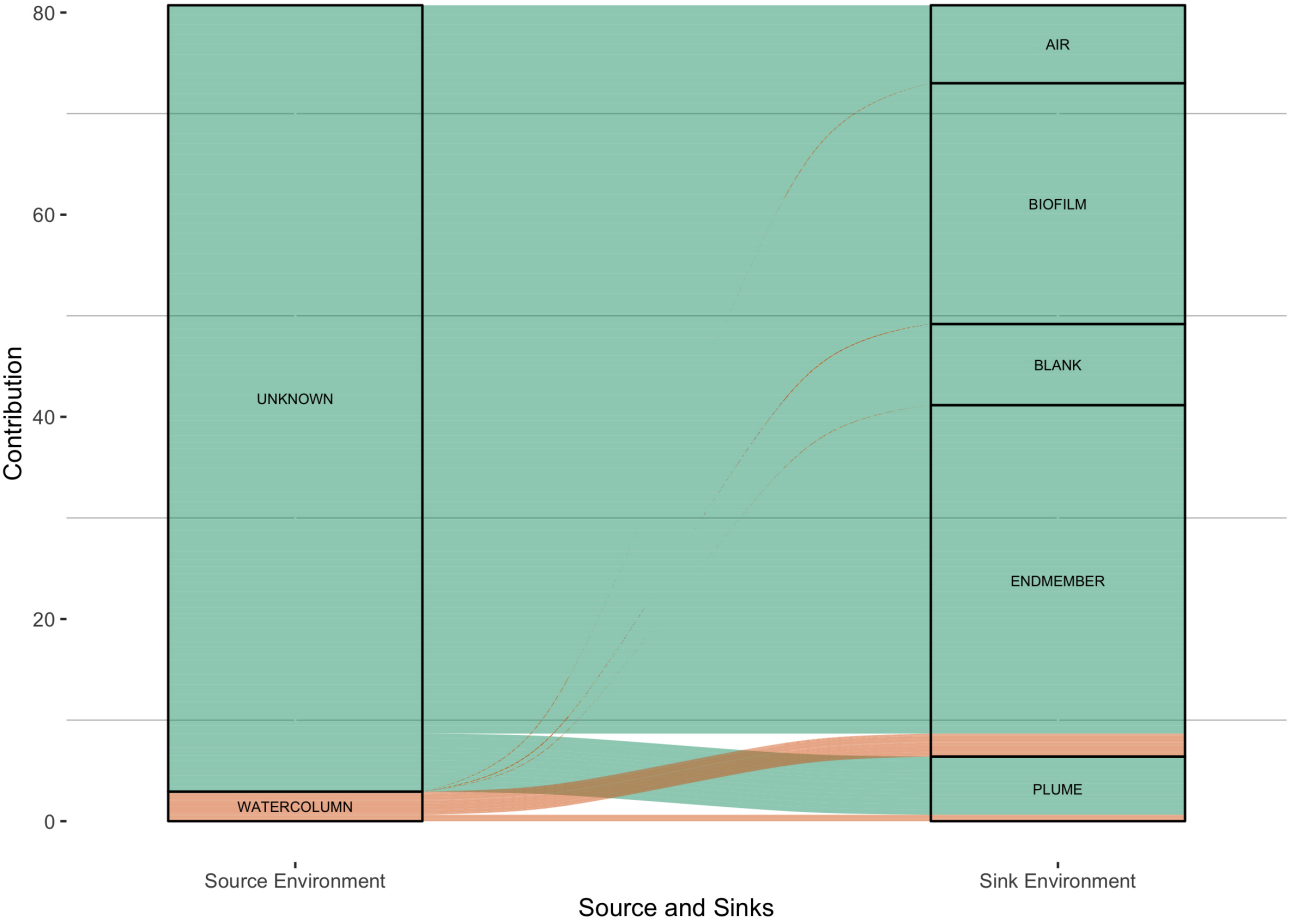
Simplified visualization of FEAST calculated source contributions of microbial community members within the LCHF local region. Sources and sinks were primarily fluid sampled historically from across the Atlantis Massif and vent end member fluids from the 2018 expedition. The left column are possible source categories into the column on the right. The majority of data cannot be assigned a source and is considered unknown, while the seawater samples collected from the water column at and around Lost City contribute notable portions into vent/endmember fluids and plume samples. Biofilm samples represent the crushed and DNA extracted carbonate samples from the 2018 expedition. Blank and Air samples were used during sample processing and DNA extraction and sequenced accordingly. Vent endmember samples were fluid samples taken directly from the venting chimneys at Lost City. Plume samples were putative Lost City plumes sampled via CTD and represent an intermixing between sea and vent environments. The data here is represented as the square root of the contribution fraction in order to enhance the visualization of small contributions against the largely unknown portions. The original unedited data can be seen in **Supplementary Table 2**.

One possible explanation for the dissimilarity within and between sites is the variable contribution from the ambient seawater microbial community. In this scenario, the least active chimneys would be more impacted by a seawater microbial imprint. To test this, source-sink dynamics were analyzed with the chimney and seawater samples collected in and around the Atlantis Massif (**Figure 1**). Seawater samples gathered away from the venting environment were considered potential microbial sources while chimney biofilms, hydrothermal fluids, and hydrothermal plumes mixing with seawater were classified as possible microbial sinks. Hydrothermal plume samples and vent fluids showed observable contributions from seawater, as expected, but chimney biofilms showed little if any contributions from seawater microbes. Only 3 of the 24 chimney samples (LC02923, LC02958, and LC02981) contained assignable seawater contribution fractions ranging from 0.02 – 0.20, one of which was an inactive vein, indicating overall, the chimney microbial community has little input from the surrounding seawater (**Supplementary Table 2**). Two of these samples have high brucite contents, and all three samples have very little calcite which is inconsistent with a simple interpretation that the microbial communities of less active chimneys become more seawater dominated. Long inactive chimneys were not included in this sampling campaign.

### Correlations between mineralogy, taxa, and genes

**Figure 2:**
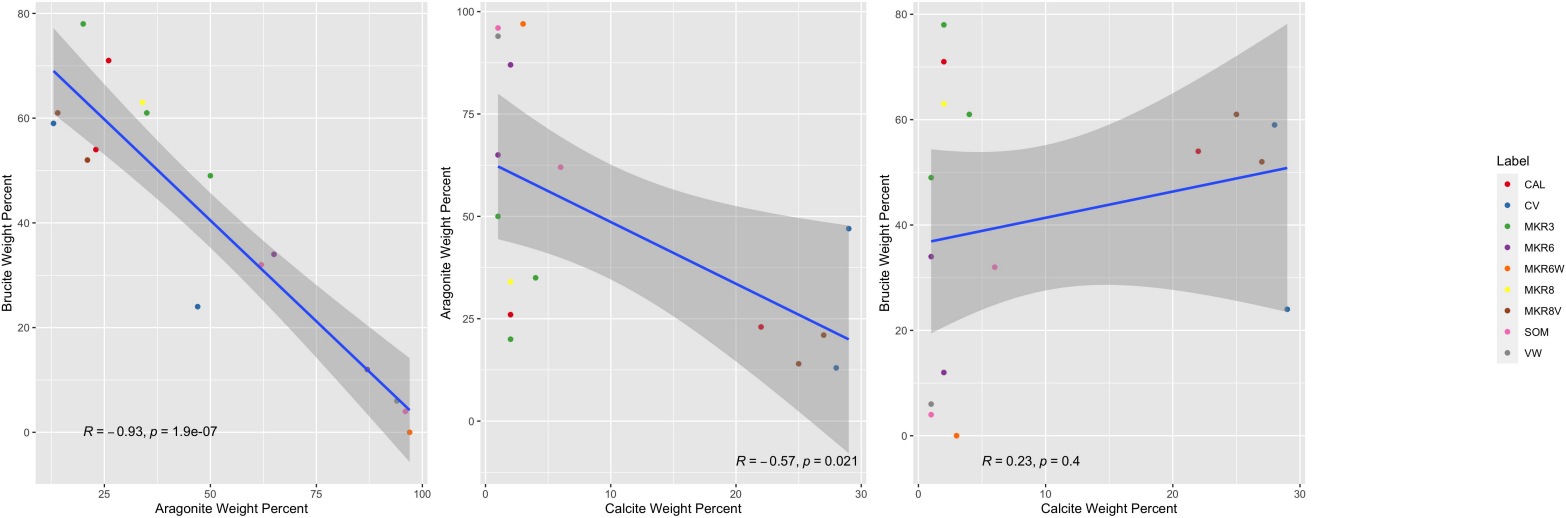
Linear correlation between the different mineralogy weight percents of samples, colored by location. From left to right: brucite weight percent to aragonite weight percent, calcite weight percent to aragonite weight percent, and brucite weight percent to calcite weight percent.

**Figure 3:**
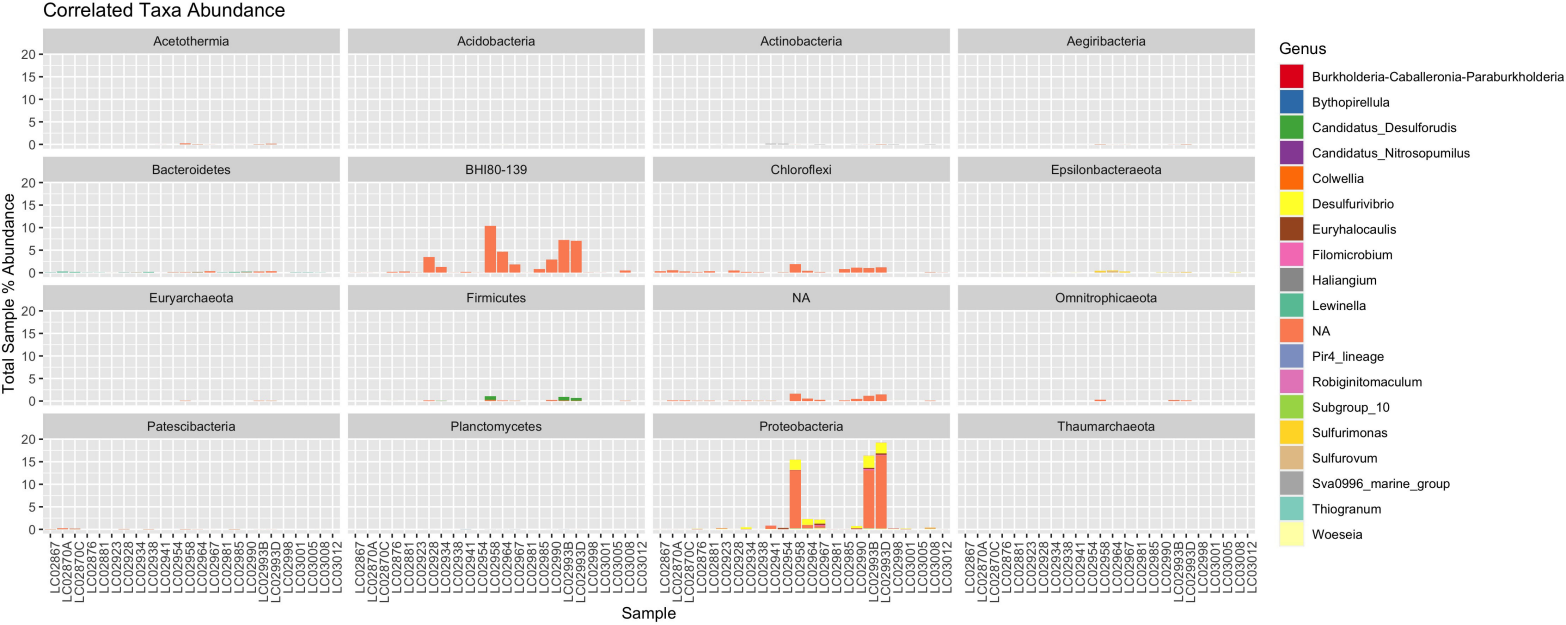
A stacked bar plot showing the abundance of correlated ASV genera in each sample, faceted by their respective phyla. Some of the phyla constitute over 15% of the total sample reads, making them significantly abundant community members.

A significant inverse correlation was noted between aragonite and brucite mass fractions (**Figure 2**). Of 10,416 microbial taxa in the ASV dataset, 86 showed significant correlations with these minerals. Taxon distributions that surpassed statistical significance (*p*<0.05, q<0.05) were grouped into ARA, BRU and CAL categories based on their associated mineral. Detailed information about these groups is available in **Supplementary Table 3** and as relative abundances in **Figure 3**. Several ASVs comprised significant fractions of the samples, upwards of 15% of total relative abundance, highlighting their centrality within their respective communities.

Clusters with a positive aragonite correlation (ARA) included 6 ASVs, 3 Proteobacteria and one each of Acidobacteria, Actinobacteria, and Bacteroidetes. The highest correlated ASV of this group (R = 0.75) belonged to Acidobacteria *Thermoanaerobaculaceae* Subgroup 10. Clusters with a positive brucite (BRU) correlation score contained 4 ASVs, a *Lewinella*, a Chloroflexi, and a Patescibacteria order MSBL5, and an unclassified bacterial sequence. The highest positive correlation score to brucite was *Lewinella* and an unknown ASV (ASV 32052 in our dataset, R = 0.66). Clusters positively correlated to calcite (CAL) contained 68 ASVs, the largest number of all three groups, and the highest correlation score of all with a *Sulfurovum* ASV (R = 0.82). 21 of the scored sequences belonged to Proteobacteria. 4 archaeal sequences were identified, 3 of which were *ANME-1b* sequences, and 1 archaeal *Candidatus Nitrosopumilus*. Interestingly, one ASV of the recently identified *Candidatus Desulforudis* had a significant association with calcite. In addition, 11 unknown bacterial sequences were moderately to very strongly correlated with calcite.

**Figure 4:**
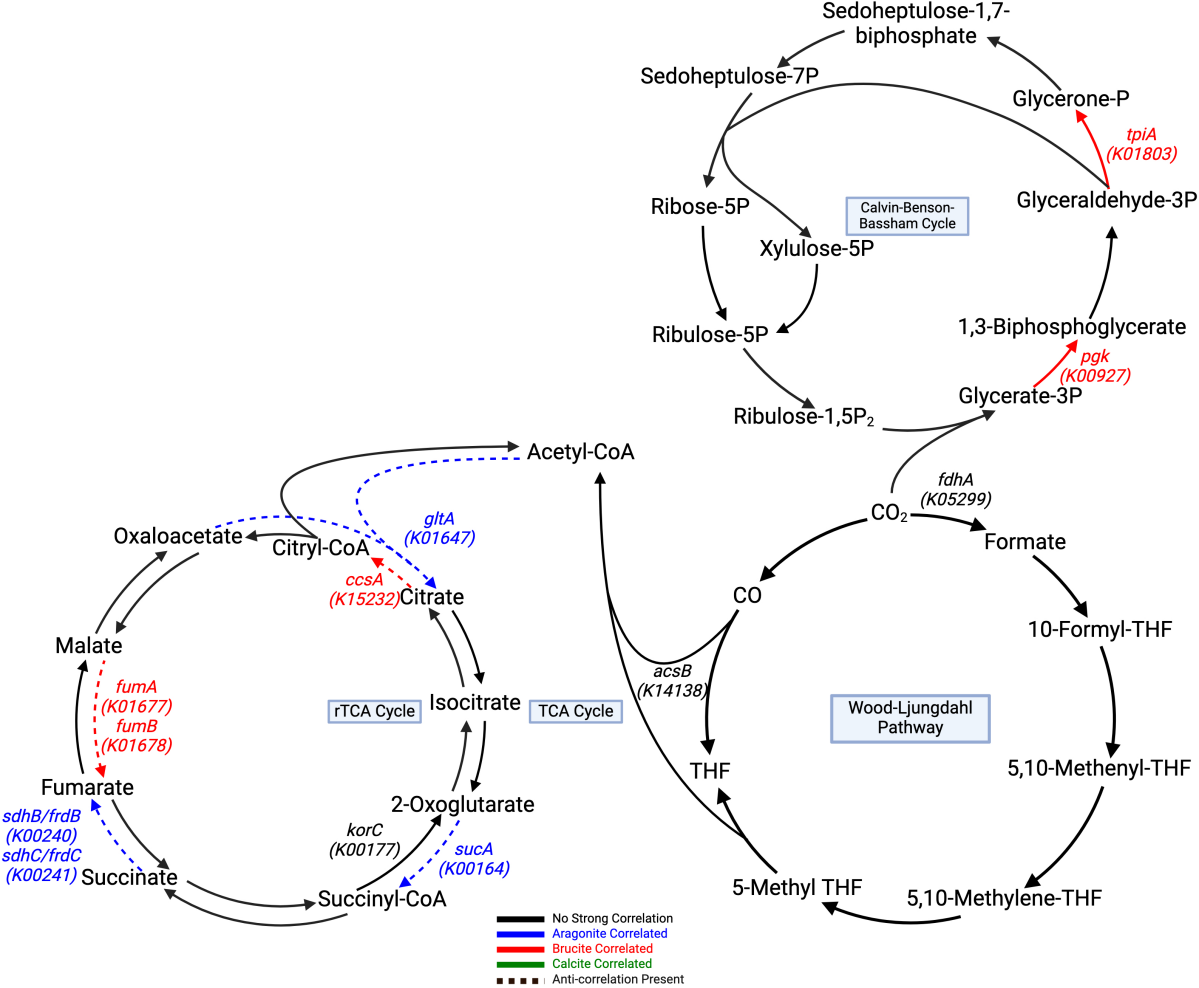
Carbon fixation pathways assembled from correlated genes with a Kegg ID color coded to their respective positive mineral correlation. A dashed line represents an anti-correlation present, as specified in **Table 3**. Genes with negative correlations are highlighted in black. Calvin-Benson-Bassham Cycle, and rTCA show generalized brucite association, while the Wood-Ljungdahl pathway shows negative aragonite correlation at key steps. The TCA cycle contains pathway steps correlated with aragonite, but negatively correlated to brucite, while the rTCA cycle pathway steps show brucite correlated genes that are also negatively correlated to aragonite. All remaining pathway genes were found but showed no significant correlations. Created with BioRender.com.

Automated annotation of individual protein sequences predicted from the LCHF chimney metagenomes identified 7,452 different genes classified by the KEGG orthology. Our analysis focused on those related to carbon, methane, nitrogen and sulfur metabolisms, as well as selected trace metal cycling components. A total of 12 genes with three carbon fixation pathways showed distinct correlations to underlying mineralogy (**Table 3**). The Calvin-Benson-Bassham cycle showed positive correlations to brucite only in the steps for the conversion of glycerate-3-phosphate to 1,3-biphopshoglycerate and from glyceraldehyde-3P to glycerone-P through *pgk* (K00927), and *tpiA* (K01803) enzyme genes (**Figure 4**).

**Table 3:** Selected significantly correlated (p < 0.05, q < 0.05) carbon cycling genes.

KEGG ID	Mineral Correlation(s)	Correlation Score	Gene	Metal Cofactor
*Carbon Cycling*				
K00927	Brucite	0.73	*pgk*	Mg
K01803	Brucite	0.68	*tpiA*	–
K14138	Aragonite	-0.72	*acsB*	Ni
K05299	Aragonite	-0.71	*fdhA*	Fe, Mo
K00164	Aragonite	0.72	*sucA*	–
	Brucite	-0.70		
K00240	Aragonite	0.69	*sdhB/frdB*	Fe
K00241	Aragonite	0.70	*sdhC/frdC*	–
	Brucite	-0.68		
K01647	Aragonite	0.67	*gltA*	Fe
	Brucite	-0.68		
K15232	Aragonite	-0.68	*ccsA*	–
	Brucite	0.68		
K01677	Aragonite	-0.72	*fumA*	Fe
	Brucite	0.72		
K01678	Aragonite	-0.74	*fumB*	Fe
	Brucite	0.72		
K00177	Aragonite	-0.67	*korC*	–
K01895	Aragonite	-0.72	*acs*	Mg, Mn
	Brucite	0.71		
K00193	Aragonite	-0.73	*cdhC*	Fe, Ni
	Brucite	0.73		
K00194	Aragonite	-0.76	*cdhD*	Fe
	Brucite	0.70		
K00197	Aragonite	-0.75	*cdhE*	Fe
K03388	Aragonite	-0.70	*hdrA2*	Fe
	Calcite	0.74		
K10946	Brucite	-0.72	*pmoC-amoC*	–
K00830	Aragonite	0.68	*AGXT*	–
	Brucite	-0.72		
K01689	Brucite	0.67	*echA*	Mg
K08692	Brucite	-0.72	*mtkB*	Mg
K14067	Aragonite	0.71	*mtkA*	Mg
	Brucite	-0.75		
K01622	Aragonite	-0.70	*K01622*	Zn

Each gene characterized by its Kegg ID displays the Pearson correlation score and if found, its metal cofactor.

The TCA and rTCA cycle genes showed some negative correlations to aragonite and brucite. In the TCA cycle, *gltA* (K01647), *sucA* (K00164), *sdhB*/*frdB* (K00240), and *sdhC*/*frdC* (K00241) positively correlated to aragonite.

**Figure 5:**
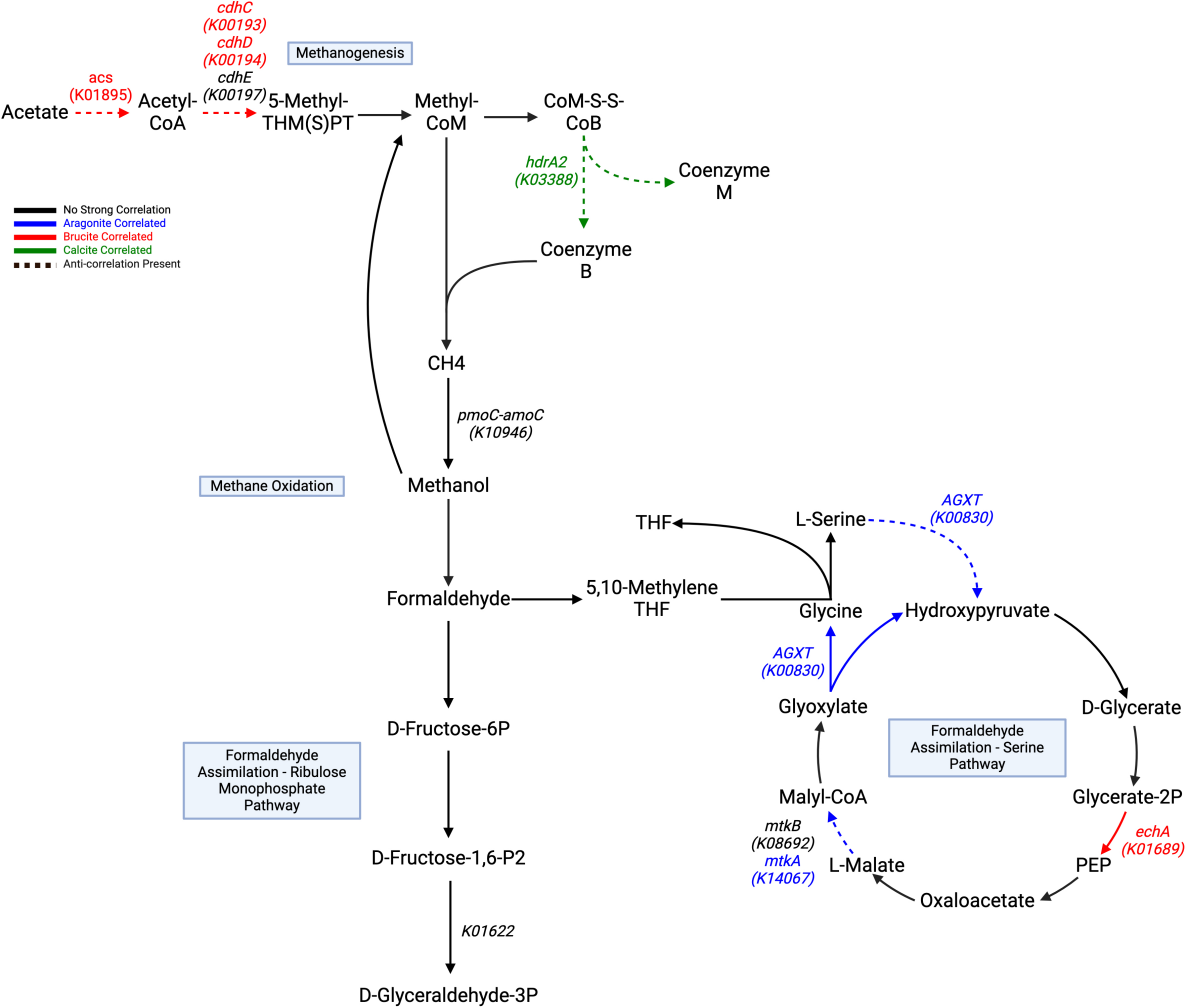
Different directions of methane/carbon cycling show trending correlations to different minerals, as highlighted in **Table 3**. Methanogenesis component genes correlate highly to brucite and calcite and showing negative correlations to aragonite. Methane oxidation and formaldehyde assimilation through the serine pathway are broadly correlate to aragonite, with negative correlations to brucite. Formaldehyde assimilation through the ribulose phosphate pathway shows no distinct positive correlation, but one strongly negative aragonite correlation for K01622. Acetate/*acs* are included to illustrate its utilization for acetoclastic methanogenesis. All remaining pathway genes were found but showed no significant correlations. Created with BioRender.com.

The Wood-Ljungdahl pathway showed no positive correlations, but *fdhA* (K05299) and *acsB* (K14138) had negative correlations with aragonite. Two subunits of CODH/ACS, *cdhC* (K00193) and *cdhD* (K00194), were positively correlated with brucite. *cdhE* (K00197) displayed a strong negative aragonite correlation. Acetyl-CoA synthetase (*acs:* K01895), which is associated with acetate metabolism in multiple pathways, including acetoclastic methanogenesis, was also positively correlated with brucite (**Figure 5**).

Methane oxidation showed no positive correlations, but *pmoC-amoC* showed a negative brucite correlation. Formaldehyde assimilation to the serine pathway contained mixed correlations, with *AGXT* (K00830), *hprA* (K00018), and *mtkA* (K14067) correlated with aragonite, and *mtkB* (K08692) negatively correlated to brucite. Conversion of glycerate-2-phosphate to phosphoenolpyruvate mediated by the *echA* (K01689) gene was correlated with brucite.

**Figure 6:**
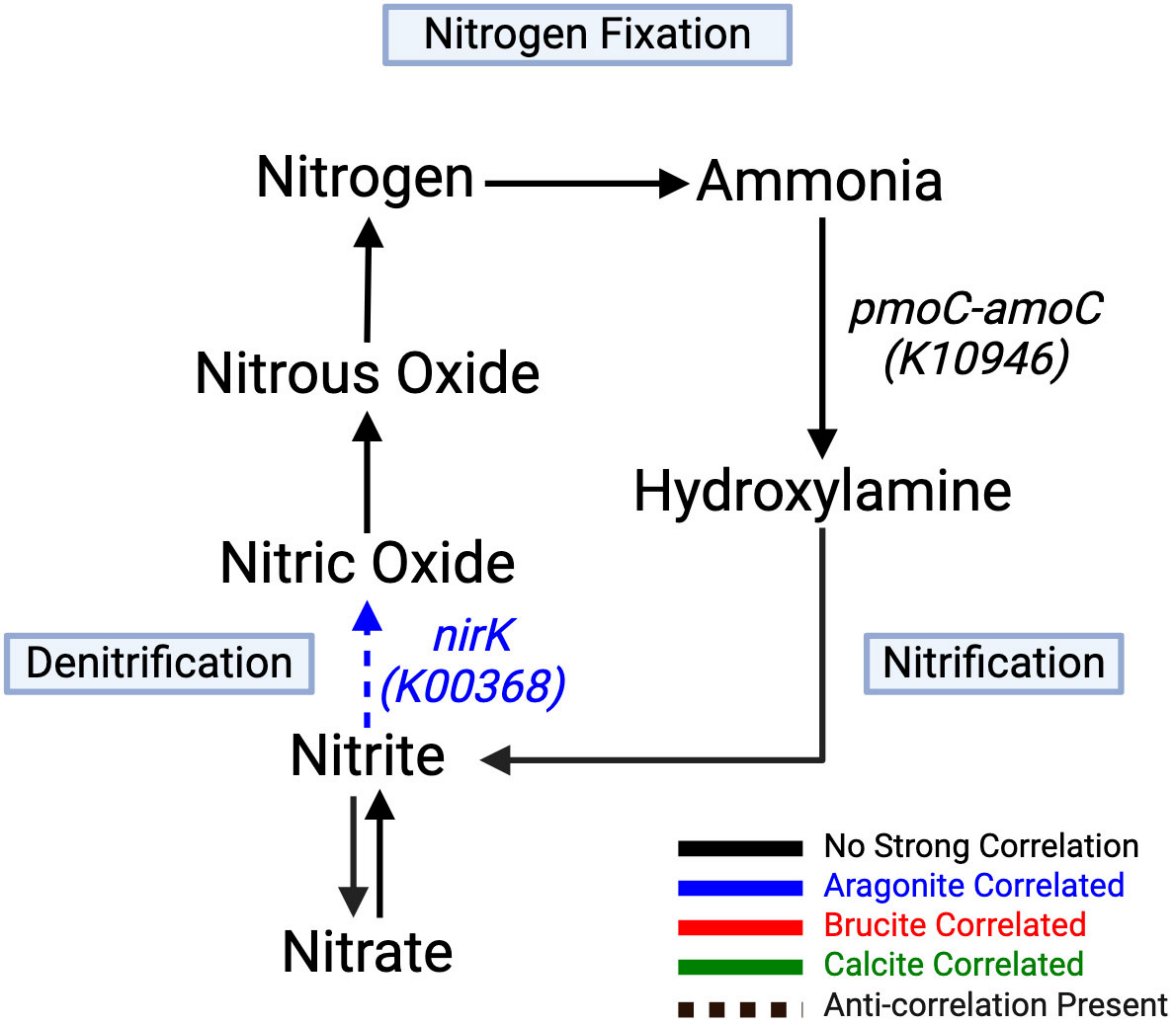
Nitrification and denitrification arms strongly correlate to aragonite. Conversion of nitrate to nitrite by the napA (K02567) gene product shows strong negative correlation to brucite. All remaining pathway genes were found but showed no significant correlations. Created with BioRender.com.

Correlations between minerals and genes involved in nitrogen cycling included those associated with nitrification and denitrification (**Figure 6**; **Table 4**). Ammonia conversion to hydroxylamine using *pmoC-amoC* (K10946) showed a negative brucite correlation, and *pmoB-amoB* (K10945) showed a strong negative brucite correlation. Denitrification from nitrate to nitrogen was aragonite-correlated, with *nirK* (K00368) showing significant negative brucite correlation.

**Table 4:** Selected significantly correlated (p < 0.05, q < 0.05) nitrogen cycling genes.

KEGG ID	Mineral Correlation(s)	Correlation Score	Gene	Metal Cofactor
*Nitrogen Cycling*				
K10946	Brucite	-0.72	*pmoC-amoC*	–
K00368	Aragonite	0.76	*nirK*	Cu
	Brucite	-0.73		

Each gene characterized by its Kegg ID displays the Pearson correlation score and if found, its metal cofactor.

**Figure 7:**
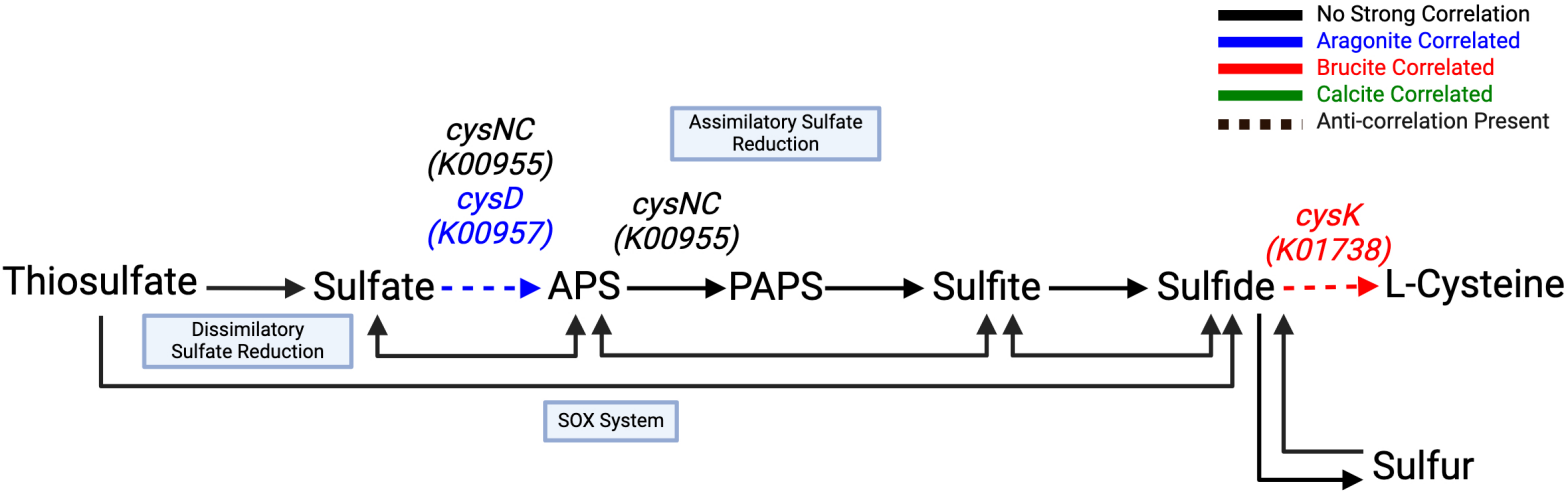
Sulfur cycling through assimilatory sulfate reduction is favored in the aragonite environment. Utilization of sulfide for cysteine biosynthesis is highly correlated to brucite via the cysK (K01738) gene product. All remaining pathway genes were found but showed no significant correlations. Created with BioRender.com.

Mineral-gene correlations related to sulfur metabolism included an aragonite correlation with *cysD* (K00957) and a strongly negative brucite correlation with *cysNC* (K00955), both of which are associated with assimilatory sulfate reduction, among other sulfur-metabolizing pathways (**Figure 7**; **Table 5**). A strong brucite correlation was found for *cysK* (K01738) with an equally strong negative correlation to aragonite, involved in utilization of sulfide in forming or maintaining the cellular cysteine pool. *hdrA2* (K03388), which is involved in sulfur redox reactions in many different organisms including methanogens and sulfur-cycling bacteria, was correlated with calcite. Components necessary for the SOX system of thiosulfate oxidation and dissimilatory processes showed no notable correlations.

**Table 5:** Selected significantly correlated (p < 0.05, q < 0.05) sulfur cycling genes.

KEGG ID	Mineral Correlation(s)	Correlation Score	Gene	Metal Cofactor
*Sulfur Cycling*				
K00955	Brucite	-0.76	*cysNC*	Mg
K00957	Aragonite	0.70	*cysD*	Mg
	Brucite	-0.82		
K01738	Aragonite	-0.69	*cysK*	–
	Brucite	0.74		

Each gene characterized by its Kegg ID displays the Pearson correlation score and if found, its metal cofactor.

A selection of genes related to trace metal transport systems were examined for any mineral associations, however all significant correlation scores were related to tungsten transport (**Table 6**). All genes exhibited negative correlations to aragonite, except for *wtpB* (K15496), which showed a negative correlation to brucite.

**Table 6:** Selected significantly correlated (p < 0.05, q < 0.05) metal transport genes.

KEGG ID	Mineral Correlation(s)	Correlation Score	Gene	Metal Cofactor
*Metals*				
K05773	Aragonite	-0.72	*tupB*	W
K06857	Aragonite	-0.73	*tupC*	W
K15496	Brucite	-0.70	*wtpB*	Mo/W
K15497	Aragonite	-0.67	*wtpC*	Mo/W

Each gene characterized by its Kegg ID displays the Pearson correlation score and if found, its metal cofactor.

### Metagenome assembled genomes and genes correlated with minerals

**Figure 8:**
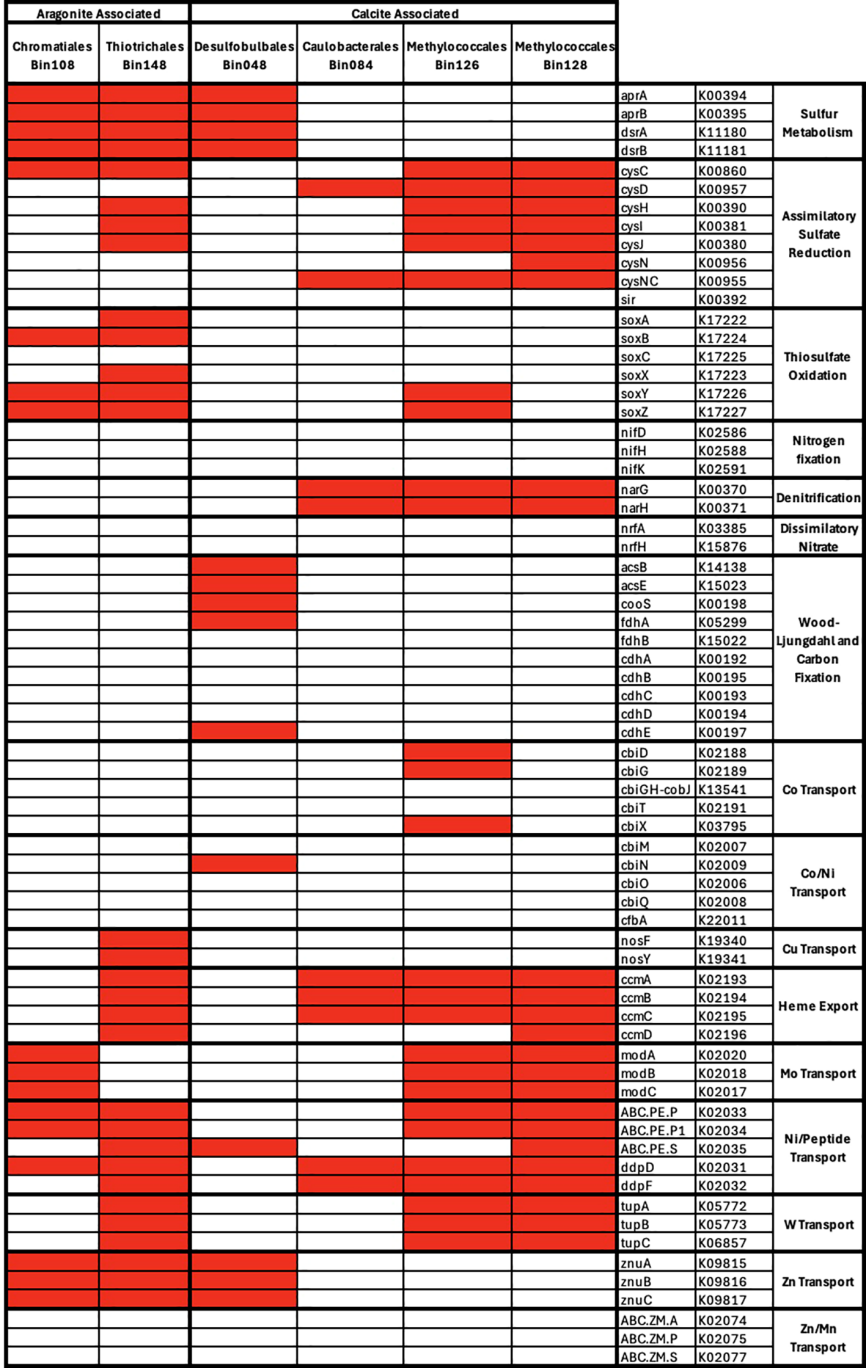
Presence and absence of selected genes related to metabolic and metal cycling in bins correlated with aragonite and calcite. Bins 90% complete with <10% contamination were selected and presented here at the taxonomic order level.

The sequence coverages (i.e. relative abundances) of MAGs with greater than 90% completion were tested for correlations with mineralogy and of these, 6 MAGs had significant correlations (p<0.05) but only one correlation (Desulfobulbales_Bin048 correlated to calcite) exhibited a q-value score better than our significance threshold (q < 0.05) (**Supplementary Table 4**; **Figure 8**). The correlations with non-significant q-value scores included aragonite correlations with two MAGs classified as Chromatiales and Thiotrichales and calcite correlations with four MAGs classified as Desulfobulbales, Caulobacterales, and Methylococcales. We will refer to these as aragonite-correlated and calcite-correlated MAGs despite the high q-value scores to survey potential overall differences between the MAGs.

The two aragonite-correlated MAGs contained genes related to sulfur metabolism, thiosulfate oxidation, and copper/zinc transport. Chromatiales_Bin108 contained genes for sulfur metabolism, molybdenum transport and zinc transport. Only one gene potentially associated with assimilatory sulfate reduction (*cysC*) and three genes for thiosulfate oxidation (*soxB*, *soxY* and *soxZ*) were present in this MAG. The combination of genes found likely represents an oxidation pathway using the *Dsr*/*Apr* genes, supported by the absence of *dsrC*, and the taxonomy of organisms with oxidative and reductive pathways associated with *dsrAB*. Two nickel/peptide permease transporters and one nickel/peptide ATP-binding protein were also found. Thiotrichales_Bin148 showed a similar pattern, with genes associated with sulfur and thiosulfate cycling, as well as complete modules for copper transport, heme export and tungstate transport. In both MAGs, there was an absence of nitrogen cycling and Wood-Ljungdahl genes.

The four calcite-correlated MAGs were classified as Desulfobulbales, Caulobacterales, and Methylococcales. Calcite-correlated MAGs contained more genes related to assimilatory sulfate reduction, denitrification, carbon cycling, and cobalt or nickel transporters. The Desulfobulbales_Bin048 contained complete modules for dissimilatory sulfate metabolism and zinc transport. A partial Wood-Ljungdahl pathway was found with the presence of *acsB, acsE, cooS, fdhA* and *cdhE.* Caulobacterales_Bin084 contained key genes for denitrification (**Figure 8**), as well as two sulfite reductase genes (*cysD* and *cysNC*) and some genes for heme export and nickel/peptide transport. The two Methylococcales MAGs had a similar collection of assimilatory sulfate reduction genes and several of the metal transport modules. However, Methylococcales_Bin126 contained two of the SOX system genes, *soxY* and *soxZ*, and several cobalt transport genes not found in its sister bin.

## Discussion

Our study used mineralogy as a proxy for varying microenvironmental conditions to investigate changes in associated microbial communities living within hydrothermal chimneys at the LCHF. The three dominant minerals at the site, aragonite, brucite and calcite, represent physical snapshots of *in situ* temperature, pH and venting conditions. Younger chimneys with active and robust flow are dominated by brucite and primary calcite and are expected to be stable with hydrothermal venting at relatively higher temperatures, while lower temperature chimneys are composed of greater aragonite concentrations (Ludwig et al., 2006; Aquino and Früh-Green, 2024a). Inactive chimneys are dominated by secondary calcite as temperatures drop below 15°C (Ludwig et al., 2006). The presence of an inverse relationship in samples between aragonite and brucite (**Figure 5**) highlights this distinct relationship, though the exact parameters of vent evolution are beyond the scope of this particular study and have been investigated by other recent studies (Aquino et al., 2022; Aquino et al., 2023; Aquino and Früh-Green, 2024a).

This pattern provides a measurable physical environmental gradient against which to analyze correlations of different microbial taxa and genes. While correlation does not imply causation, examining microbial distributions through the lens of mineral proxies provides a window into the complex chimney environments. Community analysis through taxon abundance can be confounded by multiple environmental changes and microbial turnover over long timescales, creating noise that complicates identification of significant species or consortia. Bulk analysis by nature takes advantage of a processed sample of inherently mixed and slightly different environments and microbial residents. In a site like Lost City, no two samples are exactly alike, and we are faced with the challenge in defining the strategies of a large number of distinct species utilizing different substrates under variably extremophilic conditions (Hutchinson, 1961; Aquino and Früh-Green, 2024b). The first problem, longevity of the environment, means a slow evolution of conditions over timescales longer than the biological processes, ranging from decades to tens of thousands of years, as demonstrated by the ages of active and inactive Lost City chimneys (Ludwig et al., 2006). The second problem concerns microbial turnover, resulting in a slowly evolving environment conducive to microbial coevolution and a sufficient timespan for extensive mixing and community succession. This complexity hinders the accurate identification of primary colonizers or keystone species as the community diversifies with time. This is further complicated metabolically when considering highly variable rates of microbial activity, dormancy, intercellular cooperation, inhibition, or horizontal gene transfer imparting a mosaic of functions (Price and Sowers, 2004; Edwards et al., 2012; Fuchsman et al., 2017; Colman et al., 2018; Verma et al., 2022). The resultant challenge then becomes delineating a finite set of variables suitable across the relevant environmental age that some numbers of microbes are responsive to, allowing differentiation between many of the commonly found cosmopolitan microbes and more environmentally restricted individuals. Our results conservatively focus on the most significantly correlated genes and microbes to explore the most potentially responsive relationships within a highly complex biogeochemical system. Potentially, there are many co-associations within our dataset to further explore, and this analysis represents a new perspective for consideration of the slowly changing environmental conditions at Lost City.

### Chimney microbial communities generally differ from surrounding fluids

Source tracking analysis shows that the microbial communities detected within the chimneys are not heavily influenced by surrounding seawater communities. This differs from vent fluid and plume samples showing greater exchange between seawater and subsurface fluid sources. Furthermore, the microbial community structures of chimney biofilms are both highly variable and distinct from those of venting fluids (**Supplementary Figure 4**). While aragonite remains relatively stable through extinction of hydrothermal venting, increased calcite represents the final stage of the vent lifecycle due to its formation at much lower temperatures (Ludwig et al., 2006; Aquino and Früh-Green, 2024b). Higher levels of microbial diversity and richness are observed in vents with high calcite concentration which could be related to many variables including more moderate conditions, and an increased availability of substrates and cofactors either through seawater input or their increased solubility due to more moderate pH and lower ambient temperatures. Additionally, species feeding off organic matter produced by chemolithoautotrophs may facilitate colonization of the mineral substrate by diverse consumers, akin to successional processes documented in other environments (Nemergut et al., 2007; Tarlera et al., 2008). A strong *Candidatus_Nitrosopumilus* correlation to calcite may be indicative of this gradient as they include known autotrophic ammonia oxidizers that physiologically vary accordingly between bathypelagic and hadal zones of the ocean (Qin et al., 2017; Rios-Del Toro et al., 2018; Zhong et al., 2020). The identification of correlated *Sulfurovum* ASVs firmly within the calcite associated category may be evidence of at least some taxa adapted to hydrothermal conditions, including exposure to less anoxic seawater. Previous studies examining Lost City *Sulfurovum* suggest a mixotrophic lifestyle or the capability of inhabiting a transition zone between the anoxic interior and exterior of the vent (McGonigle et al., 2020). Our results further support this hypothesis given the increased abundance of this taxon towards calcite enriched regions and the overall distribution through the vent may indeed show it colonizing an intermediate region. *Candidatus Desulforudis*, initially discovered kilometers below the continental surface and subsequently found in another terrestrial serpentinite-hosted site, suggest potential interconnections between subsurface environments and the possibility of an ecological niche along the venting pathway or a subsurface-driven dispersal mechanism (Sabuda et al., 2020; Gittins et al., 2022). *Sulfurovum* and multiple other hydrothermally associated taxa have been frequently identified in marine sediments to varying extents further highlighting some shared environmental similarity between different locales where they are found (Mino et al., 2014; Anderson et al., 2017; Mori et al., 2018; Sun et al., 2020).

Microbial activities have also been known to be nucleation sites for carbonate precipitation and have been hypothesized to contribute to the carbonate formation in hypersaline lakes, and low temperature alkaline hydrothermal vents such as in Prony Bay (Dupraz et al., 2004; Ries et al., 2008; Pisapia et al., 2017). There is a high likelihood that carbon metabolism increases carbonate mineralization either through carbonic anhydrase (catalyzing bidirectional formation of bicarbonate from CO_2_ and water) or urease (catalyzing urea breakdown to CO_2_ and ammonia) activity, with additional evidence previously showing interconversion between formate and CO_2_ (Wei et al., 2015; Krause et al., 2018; Lang et al., 2018; Sundaram and Thakur, 2018). This possible interlinkage between CO_2_ cycling and carbonate precipitation could explain the microbial correlations with aragonite and calcite. The most abundant taxa, *Thiomicrorhabdus* (111 ASVs), is known to contain carbonic anhydrase activity, and although it is not correlated with specific minerals in our dataset, it is widely dispersed and occupies much of the carbonate-bearing substrate within the site (Brazelton and Baross, 2010; Brazelton et al., 2010). The same can be found for *Desulfotomaculum* (50 unique ASVs)*, Sulfurovum* (74 ASVs) and *Woeseia* (85 ASVs), and if the pattern holds true, would place them within intermediate regions of the vent at Lost City and associated with a wide habitat range on the seafloor.

The large number of ASVs associated with the most abundant taxa points to a microdiversity arising from long lived and relatively stable populations within the site. The higher resolution provided with ASV analysis highlights the possibility that large ASV populations contain slightly different ecotypes across an evolving habitat range as a persistence and resilience strategy of the taxa, similar to environments with seasonal cycles (Larkin and Martiny, 2017; García-García et al., 2019; Fodelianakis et al., 2022). Though Lost City does not experience significant seasonal changes, the venting environment does change over the lifetime of the vent and the microdiversity could be a byproduct of this slow change at the microenvironmental scale over time (Martiny et al., 2023).

### Brucite relationships as a window into the most extreme metabolic adaptations

The very limited number of taxa correlated with brucite, including the lack of any correlated high-quality MAGs, may be indicative of the more extreme environmental conditions associated with brucite stability. Aragonite and brucite show an inverse correlation to each other (**Figure 2**), and some taxa mirror this distribution creating the appearance of distinct habitat ranges for the correlated taxa. *Lewinella*, for example, is prevalent and diverse in anaerobic and saline environments, including bioreactors (Mourgela et al., 2023). An ASV of the order MSBL5 correlated with brucite and is notable because of its identification in hypersaline and other serpentinizing environments, such as the Prony Bay Hydrothermal Field (Suzuki et al., 2013; Postec et al., 2015; Frouin et al., 2018; Lecoeuvre et al., 2020). Several other MSBL5 ASVs were associated with calcite, and when considering the formation of primary calcite with brucite early in vent formation, and association with other serpentinizing sites there is a possibility that this order’s niche may be within the interior of the vent brucite/calcite regions. A similar pattern can be seen for ASVs of class Parcubacteria, known to exist in hyperalkaline and hadal environments, for which there is a single brucite associatedtaxa, and two calcite associated ones (León-Zayas et al., 2017; Suzuki et al., 2017).

### Gene correlations highlight metabolic differentiation within vent chimneys and may inform community differentiation

Within vent chimneys, gene correlations shed light on the metabolic differentiation among resident microbes. By mapping correlated genes within their respective pathways, we highlight the dominant metabolic strategies of these microbial populations attributable to their environment and location within LCHF. Our amplicon sequence data and observed high number of ASVs within different taxa suggests specialized populations adapted to various niches within the Lost City environmental range. Substrate availability also plays a pivotal role, influencing either an increase in gene abundance within specialized microbial populations over time or reflecting inherent limitation *in situ*. Limited and essential substrates necessitate the heightened production of uptake and utilization machinery, leading to increased gene abundances associated with these processes.

While we identified few correlated components of the TCA and rTCA cycles (**Figure 4**), the former exhibited more correlations with aragonite. Initial use of acetyl-CoA by *gltA* correlated with aragonite, but also strongly negatively correlated with brucite. The rate-limiting step of the TCA cycle involves the conversion of isocitrate to 2-oxoglutarate/α-ketoglutarate, and its conversion to succinyl-CoA correlates with aragonite but negatively with brucite. The positive correlations observed for *sdhB/sdhC* (succinate dehydrogenase complexes) may represent an adaptation for maintaining TCA cycle products, particularly fumarate, under stressful conditions which would correspond with moderate hydrothermal conditions in high aragonite domains (Gaupp et al., 2010). Here, we presume that similar correlations in a common and critical cycle as this may indicate the overall trend, and why their placement with the strong negative and positive correlations places them in an aragonite region. Though only two correlated products were identified, the rTCA cycle may show the reverse relationship. Two genes positively correlated with brucite and negatively correlated with aragonite were *fumA* and *fumB*, which encode for conversion of malate to fumarate, further underlining convergence on fumarate. Two genes independently correlated to brucite may be driven by the specific stability of a final two subunit product over a larger single enzyme, but it is unclear why. The large subunit of citryl-CoA synthetase encoded by *ccsA*, highly correlating with brucite, participates in the specific ATP-dependent conversion of citrate to citryl-CoA, and has resemblance to succinyl-CoA synthetase with a high degree of ATP sensitivity (Aoshima et al., 2004). An inhibitory effect by succinyl-CoA synthetase may cause discontinuation of the rTCA cycle and would explain why an increased abundance of *ccsA* would help drive the conversion process forward against a possible inhibitory threshold. The mixtures of negative and positive correlations here could be suggestive of key cycle components being brucite associated.

Because of the prevalence of archaea associated with methanogenesis and the anaerobic oxidation of methane in LCHF fluids and chimneys we were particularly interested in testing for mineral correlations with genes involved in methane metabolism and the Wood-Ljungdahl pathway (Schrenk et al., 2004; Brazelton et al., 2006; Brazelton et al., 2022). Some of the subunits of the CODH/ACS enzyme (*cdhC*, *cdhD*, and *cdhE*) exhibited correlations with aragonite or brucite, but overall, there were no consistent mineralogical correlations with the Wood-Ljungdahl pathway. One likely explanation for this result is the presence of Wood-Ljungdahl genes in several different archaeal and bacterial species in LCHF fluids and chimneys (McGonigle et al., 2020; Brazelton et al., 2022).

Multiple bacterial species associated with aerobic methane oxidation have been identified in LCHF chimneys and fluids (Brazelton et al., 2006; Brazelton et al., 2022). Indeed, our results show that brucite has a strong negative correlation with *pmoC-amoC*, consistent with aerobic methane oxidation occurring at cooler, more seawater-dominated zones of the chimney, leaving methanotrophy as a regional function isolated from the primarily reducing conditions at the vent core. Furthermore, the ribulose monophosphate pathway to D-fructose-6P associated with type I methanotrophs showed no clear correlations, while type II methanotrophs utilizing the serine pathway of formaldehyde assimilation showed strong negative correlations to brucite (Bowman, 2006).

### Trace metal availability and sensitivity can influence key metabolic cofactors

Many of the key enzymes in carbon fixation and sulfur, nitrogen, and methane cycling rely on metal cofactors for the catalytic centers of their products. Lost City chimneys, however, are considered iron-poor with concentrations below a 50 ppm detection limit, and other metals (Mn, Ni, Co, Cu, and Mo) are present in single digit ppm concentrations or below detection levels in active vents (Ludwig et al., 2006). Therefore, we were interested in potential connections between metal transporters and specific minerals. We identified no correlations with iron transporters or related genes, possibly indicating no significant differences in iron availability at the site. However, genes related to tungsten or tungsten/molybdenum transport show a strong correlation with brucite. Where those metal-associated genes show relationships could indicate a localized metal requirement and a significant energetic expenditure for their uptake. Previous studies examining the distribution of trace metals between biofilm and chimney mineral matrix showed 83% of iron detected was concentrated within the biofilms, and the remainder in the chimney matrix, in addition to high levels of tungsten accumulation in hydrothermal environment (Demina et al., 2015; Coimbra et al., 2017). The correlations point to a possibly increased utilization of tungsten and/or molybdenum within brucite regions (Ludwig et al., 2006).

## Conclusions

Our research focused on using mineral abundances as a physical indicator of microenvironmental conditions within Lost City chimneys. This approach allowed us to pinpoint microbial taxa and crucial metabolic components that are responsive to these mineralogical variations. Initially, vent chimneys are rich in brucite, reflecting the hot, anoxic fluids from the serpentinizing system below. In this harsh setting, only a few organisms persist, possibly relying on mutually beneficial relationships to make the most of the available methane, hydrogen, and limited dissolved inorganic carbon. As the vents mature, they develop a combination of aragonite and brucite, with brucite diminishing as temperature decreases. This shift leads to a less exclusive environment, supporting a wider variety of microbes and metabolic processes, as evidenced by changes in carbon fixation, and methane, nitrogen, and sulfur cycling. In the absence of cultured specimens, this kind of high-throughput data analysis helps us to dissect how these microbial communities are structured and to identify their key constraints. Lost City-type environments are believed to have been widespread on early Earth. Studying the structure of microbial communities in these environments can reveal how they, and their potentially ancient metabolic strategies, evolved with the changing chemistry of the system. The origins of protometabolism on Earth may have occurred in conditions like those at Lost City (Russell and Hall, 1997; Sojo et al., 2016; Kitadai et al., 2019). This implies that the advantages and limitations of this environmental setting have persisted for billions of years, with contemporary organisms highly adapted to them. By further pinpointing, measuring, and defining the conditions these microbes persist within, we can gain a clearer understanding of how microbial life endures in other harsh environments.

## Data Availability

The datasets presented in this study can be found in online repositories. The names of the repository/repositories and accession number(s) can be found below: https://www.ncbi.nlm.nih.gov/, PRJNA1072998 https://www.ncbi.nlm.nih.gov/, PRJNA1074139.
