# Supplementary material for: Microbial community differentiation in vent chimneys of the Lost City Hydrothermal Field reflects habitat heterogeneity
Source: Front Microbiomes. 2025 Jan 20;3:1401831. doi: 10.3389/frmbi.2024.1401831 (PMC12993503; doi:10.3389/frmbi.2024.1401831)

SI Figure 1

Figure 1 - Bray-Curtis dissimilarity NMDS of the microbial composition between samples from Lost City vents. Circles are individual samples colored according to their respective locations, labeled according to originating vent as compiled in Table 1.

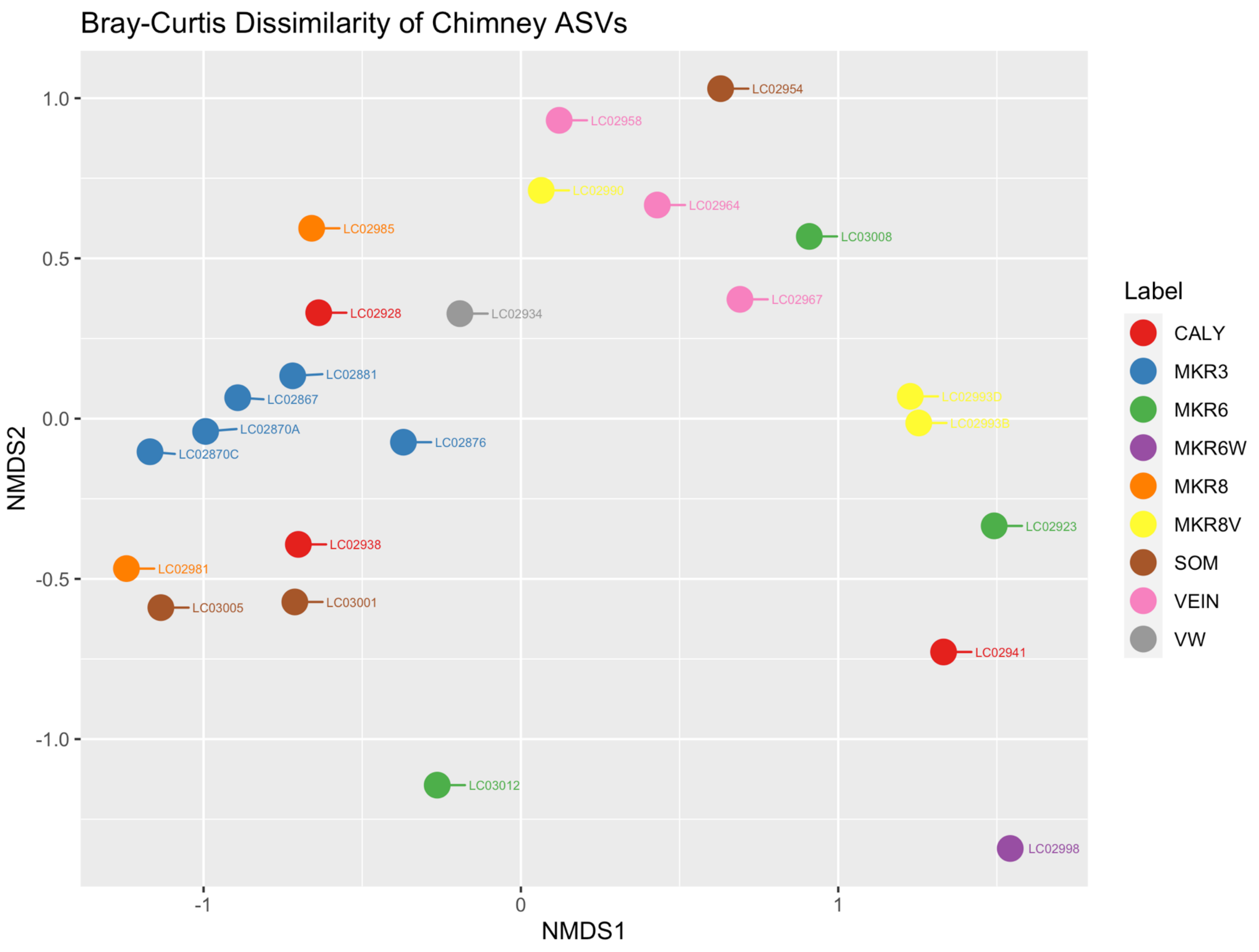

SI Figure 2

Figure 2 - Community richness / alpha-diversity comparing observed, Chao1, ACE, and Shannon indices for each location based on the untrimmed ASV counts.

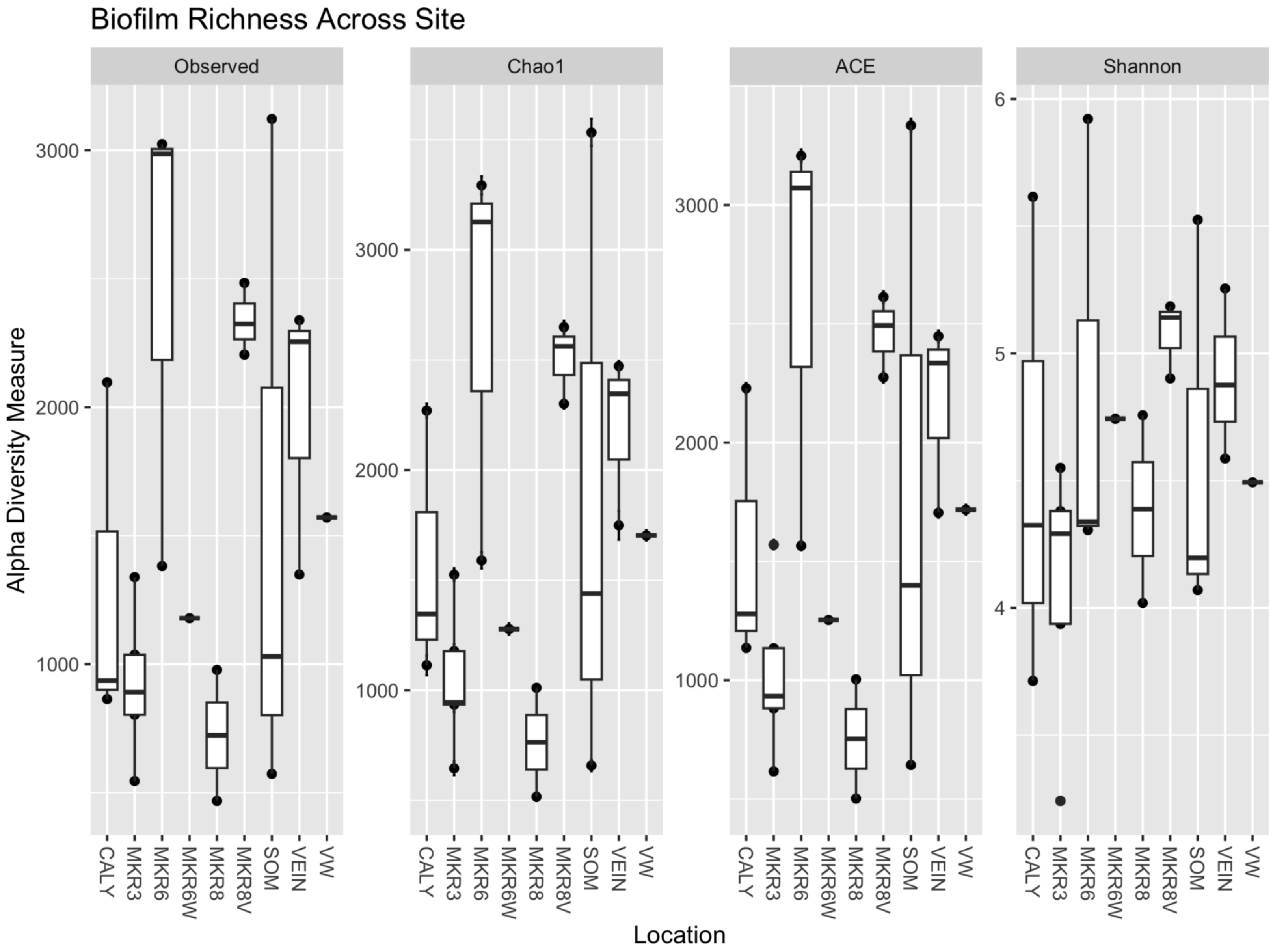

SI Figure 3

Figure 3 - Percent relative abundance of the top 50 ASVs’ genera from chimney biofilms by genus within each sample.

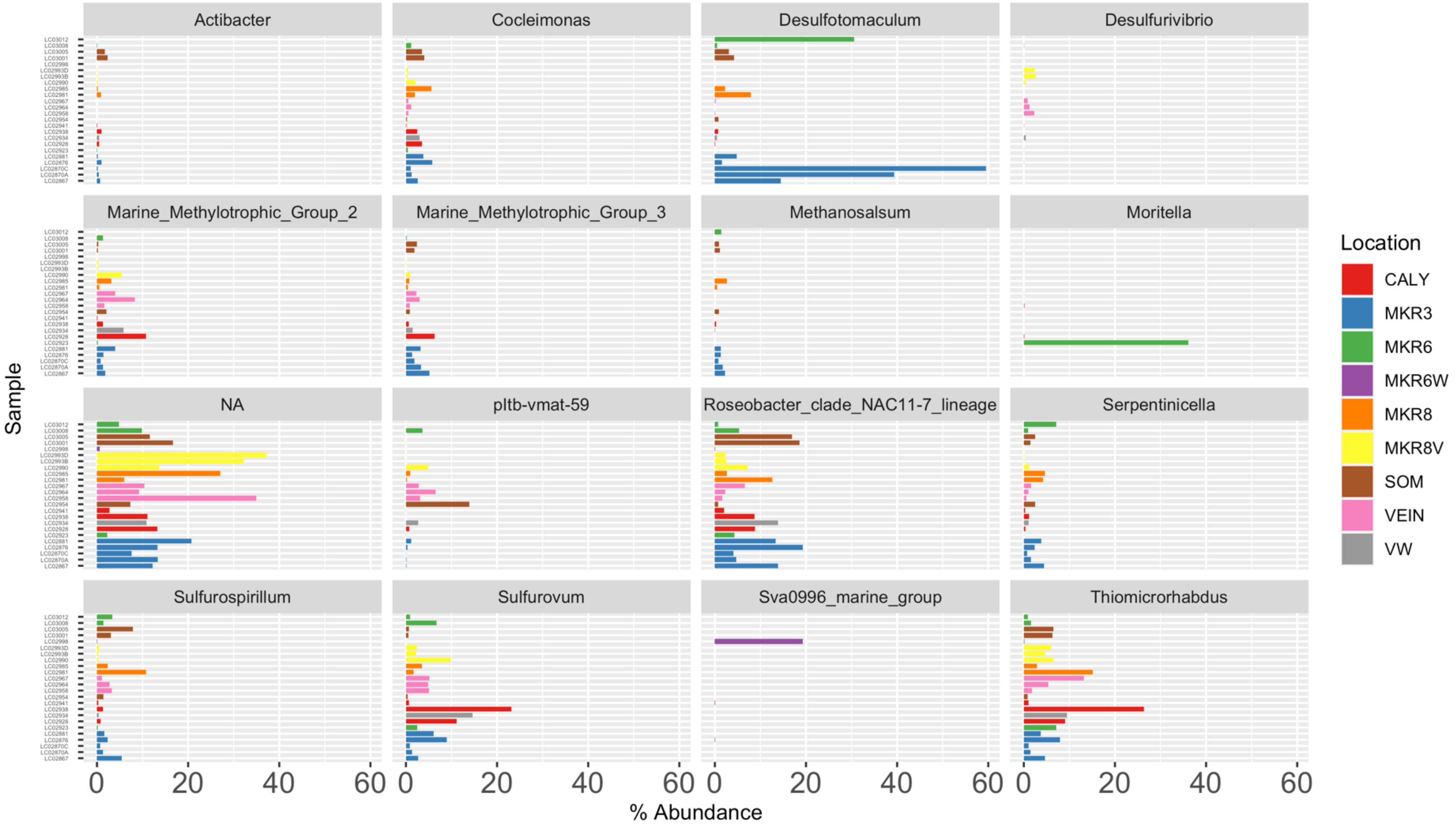

SI Figure 04

Figure 4 – NMDS plot encompassing all the fluid data gathered from Lost City vents, the Atlantis Massif and IODP sampling in addition to seawater showing a large degree of variation within chimney samples that also differentiates them significantly from fluids.

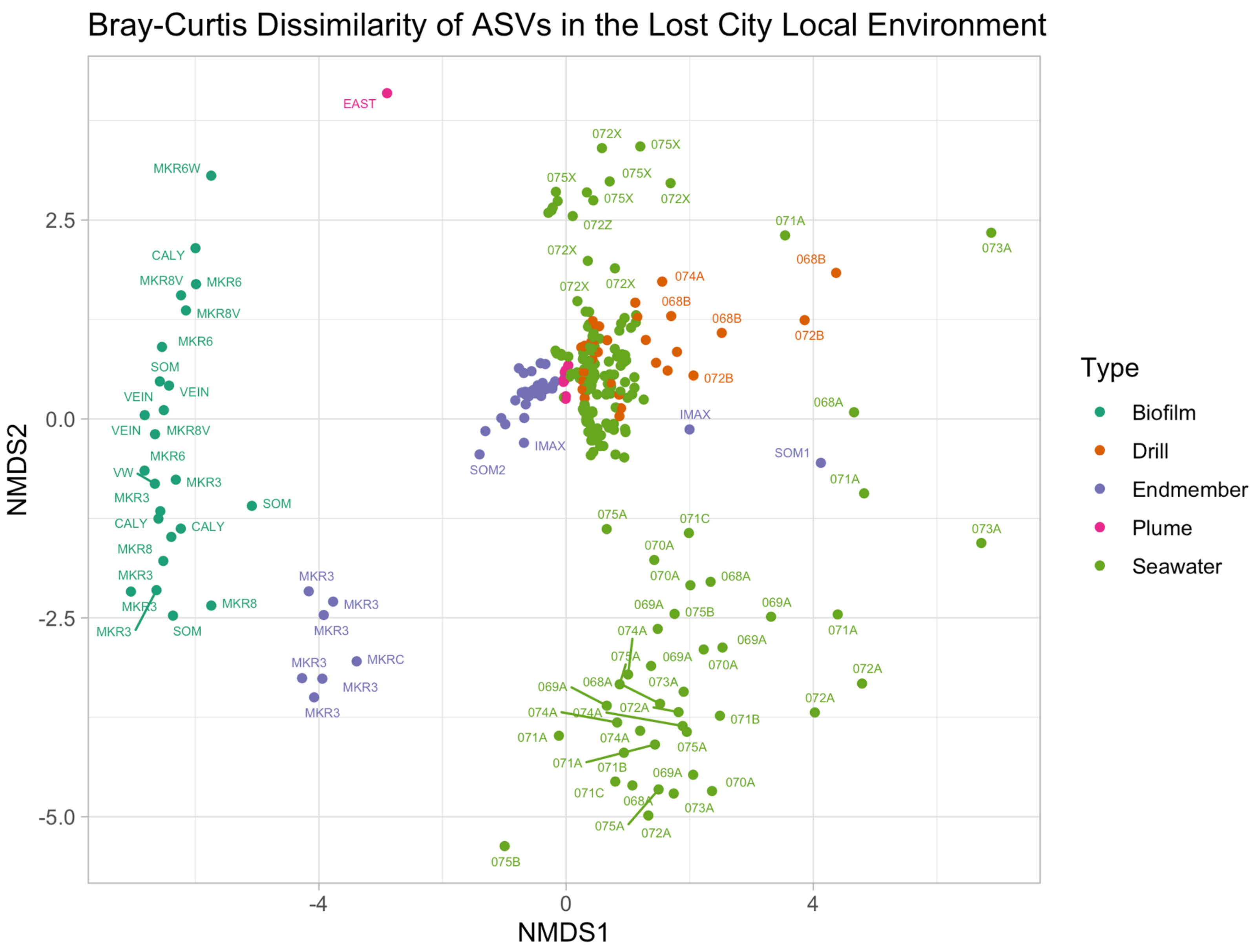

Supplement: Supplementary file 5 [file DataSheet1.pdf]
